# How is the way we spend our time related to psychological wellbeing? A cross-sectional analysis of time-use patterns in the general population and their associations with wellbeing and life satisfaction

**DOI:** 10.1186/s12889-021-11712-w

**Published:** 2021-10-14

**Authors:** Samuel Tomczyk, Laura Altweck, Silke Schmidt

**Affiliations:** Department of Health and Prevention, University of Greifswald, Robert-Blum-Str. 13, 17489 Greifswald, Germany

**Keywords:** Quality of life, Cluster analysis, Life style, Mental health, Public health

## Abstract

**Background:**

Time-use surveys can closely monitor daily activities, times of stress and relaxation, and examine predictors and trajectories with regard to health. However, previous studies have often neglected the complex interaction of daily activities when looking at health outcomes.

**Methods:**

Using latent profile analysis, this study examined patterns of self-reported daily time use (0–12h hours) for nine types of behaviour (work, errands, housework, childcare, care of persons in need, education, repairs and gardening, physical activity, and hobbies/leisure-time activities) in the 2018 wave of the German Socio-Economic Panel (*N* = 30,152; 51.9% female; *M* = 46.87 years). Sociodemographic variables, affective wellbeing, general and domain-specific life satisfaction, and self-rated health were inspected as predictors via multinomial logistic regression models.

**Results:**

Six latent profiles emerged: full-time work (47.2%), leisure (33.8%), childcare (8.9%), education (7.0%), part-time work & care (2.6%), and care (0.5%). Overall, the care and part-time work & care profiles showed the lowest wellbeing scores, lower subjective health, and life satisfaction. Women were more likely to be members of the care and childcare profiles. Men were more likely to belong to the full-time work profile, and they reported significantly higher wellbeing than women.

**Conclusions:**

The analysis revealed distinct patterns of time use and a burden on women, given their investment in care and childcare. Part-time work, and care seemed particularly demanding, and thus, are important areas for prevention, for instance, regarding mental health problems. However, time use was assessed via self-reports, therefore future studies could implement objective measures like digital trackers to validate findings.

**Supplementary Information:**

The online version contains supplementary material available at 10.1186/s12889-021-11712-w.

## Background

How we spend our time determines every aspect of our life and is deeply intertwined with our sociodemographic background: a single parent, working full-time, for instance, is more likely to spend time on work, childcare and less likely to allocate time on personal hobbies compared to a student or a pensioner. From a health research perspective, time use surveys have the potential to illuminate individual times of stress and relaxation and thus uncover unique opportunities for preventive intervention [1–4]. Yet, previous studies have often neglected the complex interaction of daily activities [5–7], for instance, the complementary function of physical activity at the end of the workday and its impact on wellbeing. This then raises the question: can time also equal health? To examine this association, we focus on the interdependency of several areas of daily time use (e.g., work, childcare, education, sports), with respect to sociodemographic differences, and their connection to psychosocial health [8, 9].

### Physical activity, and sedentary behaviours

An association between daily physical activity, sedentary behaviours, and health has been established [5, 10, 11]. For instance, time spent on physical activity, like in sport clubs, is positively associated with health and wellbeing, whereas screen-based and sedentary behaviours are often linked to poor health outcomes [5, 10, 11].

### Paid work

In general better health status is seen in full-time versus part-time or not working individuals [12, 13], but long working hours are related to psychosomatic health complaints [14], onset of depression [7], and poor self-rated health [15]. This strongly affects men who tend to assign greater importance to work than women [16], which can be explained by internalized traditional gender roles that frame men as more oriented towards agency/achievement (e.g., work), and women towards communality/relationships (e.g., childcare) [17].

### Unpaid work (e.g., childcare, housework)

Van der Meer [18] found that while men gain most of their status from their job, women attain their status from numerous sources. Men with children who lose their paid work are more likely to see it as a defeat while women are more likely to see it as an opportunity [19]. In a recent study of health trajectories across the lifespan, women’s health was more closely linked to aspects of family than paid work, and non-traditional lifestyles (i.e., not working full-time, having a steady partner or children) were associated with worse physical health in men but not in women [20]. In general, women seem to be less conflicted about their dual commitment to work and family (e.g., childcare) than men [21, 22]. However, mothers report less happiness, more stress and greater fatigue than fathers when asked about time spent with their children [23]. Beyond childcare, if a relative or close person is unwell, it also mainly falls to women to take care of them; although both male and female carers report worse mental health than non-carers [24, 25]. Interestingly, housework appeared to be a stress relief to female but not male caregivers [25]. This interconnectedness of daily activities surrounding work, family, and leisure time led us to inspect more complex patterns of daily time use in the general population.

### Time-use profiles

We found one study that examined daily time-use profiles [26], based on the American time use survey [2]. However, this study recoded daily activities as necessary, committed, contracted, and free, which shifts the focus from an individual to a social level of analysis, since the activity is coded according to its social function. The authors report eight different latent classes of daily time use that are differentially associated with sociodemographic data, for instance, shorter contracted time (e.g., paid work) in women and elderly people, and more committed time (e.g., household chores) in parents [26]. The study illustrates the nuances of time structures in daily life, and its sociodemographic determinants. However, it does not elaborate on the associations with wellbeing and mental health, which we consider essential in epidemiological and prevention research to identify risk factors and develop tailored interventions to foster positive mental health in the population, particularly regarding health equity and gender [21, 22]. Therefore, this study aims to:

1. Determine patterns of daily time use in an adult population sample via latent profile analysis.
2. Examine the association of psychological wellbeing with daily time-use patterns controlling for sociodemographic factors.
3. Assess the interaction effects of gender and psychological wellbeing in relation to time-use patterns.

## Methods

This study is based on data from the Core-Study of the German Socio-Economic Panel (GSOEP), an annual representative longitudinal study of private households, which started in 1984 [27, 28]. We used data from the most recent wave of data collection in 2018 and restricted the sample to an age of 18–100 years. This study is a secondary analysis of GSOEP data; therefore, no additional ethical approval was needed. Information on data collection procedures and ethical approval of the primary study can be found elsewhere [27, 28]. The research was approved by the appropriate ethics committee and conformed to the principles embodied in the Declaration of Helsinki (see also Ethics approval).

### Variables and measures

*Daily time use* was measured with the question “What is a typical weekday like for you? How many hours per normal workday do you spend on the following activities?”. Activities included 1) *work*, apprenticeship (including travel time to and from work), 2) *errands* (shopping, trips to government agencies, etc.), 3) *housework* (washing/cooking/cleaning), 4) *childcare*, 5) *care and support of persons in need of care*, 6) *education* or further training (also school/university), 7) *repairs* on and around the home or car, and *garden* or lawn work, 8) *physical activity* (sport, fitness, gymnastics), and 9) hobbies and other *leisure*-time activities. We recoded time-use variables to reflect a continuum of 0 to 12+ daily hours per domain. This cut-off was selected to represent a regular workday (i. e., 8 h) plus leisure time to allow for a variety of daily activities.

*Self-rated health* was measured using the item “How would you describe your current health?”. The 5-point scale from *1*(very good) to *5*(bad) was inverted so that higher values reflected better self-rated health.

*Affective wellbeing* was measured with the question “Thinking back on the last four weeks, please state how often you have experienced each of the following feelings. How often have you felt ...” on a 5-point scale (*1*[very seldom] to *5*[very often]) [29]. One item measured a positive emotion (“happy”) and the other three items measured negative emotions (“annoyed”, “afraid”, “sad”). The negative items were reverse-coded and summed with the positive item, so that higher values reflected greater affective wellbeing.

*General* and *domain-specific life satisfaction* was measured using the items „How satisfied are you with …” life overall, your health, your sleep, your leisure time, and your family life [30]. An 11-point scale of *0* (completely dissatisfied) to *10* (completely satisfied) was used, so that higher values reflected greater satisfaction.

*Sociodemographic variables* comprised *age* (years), *household income* (Euros), *number of children living in household*, *employment status* (dummy coded: no employment^1^ [reference], part-time employment, & full-time employment), *region* (1 = East Germany, 0 = West Germany), *gender* (1 = female, 0 = male), *education* (ISCED-11 dummy coded: low [1–3] (reference), medium [4, 5], & high [6, 7, 10]), and *marital status* (1 = married, 0 = not married).

### Statistical analysis

We examined missing data and distributions of the analysis data set by calculating mean values, standard deviations for continuous variables, and relative frequencies for categorical variables. Data was compared between genders (male, female) via t-tests, ANOVA, and chi square tests using the software *R* version 3.6.2 [31]. The R library *mice* was used for imputation of missing data [32].

*Latent profile modelling* of daily time-use patterns was performed with Mplus 7 [33] using robust maximum likelihood estimation and 500 sets of random start values. The estimation process started with two latent profiles, the number of latent profiles was increased to six, whilst comparing model fit [34, 35]. Overall model fit was tested with the bootstrapped likelihood ratio test (BLRT) that compares the estimated model to a model with one less class: a significant value indicates better fit for the estimated model. Parameter sparseness was indicated by the Akaike Information Criterion (AIC) and the sample-size adjusted Bayes Information Criterion (SSABIC), with a lower value indicating greater sparseness. Classification quality measures comprised average latent class probabilities (ALCP), and entropy. The closer to 1 the better the fit; an entropy of at least .7 is recommended [34]. Finally, theoretical tenability refers to the validity and plausibility of latent profiles considering the literature and the theoretical background. To consider the impact of age as a proxy of retirement status on daily time use patterns, we performed sensitivity analyses by examining latent profiles in two subsamples, aged 18–65 years (*n* = 25,048), and aged 66+ years (*n* = 5104). Latent profile modelling followed the same steps as described above.

*Multinomial logistic regression models* (MLRs). In MLRs, we predicted the identified time-use profiles by sociodemographic variables, psychosocial health indicators, and interactions of health indicators and gender. MLRs were calculated following the three-step approach using the r3step command in Mplus [36].

## Results

### Descriptive statistics

The sample comprised 30,152 persons (51.9% female), with a mean age of 46.87 years (*SD* = 17.61). The majority (see Table 1) had a low level of education and was currently not employed or retired but reported rather high levels of wellbeing and life satisfaction (e.g., an average around 7 on a scale from 0 to 10). Most time was spent on work (4.6 h/day), followed by leisure activities (1.77 h/day), childcare (1.60 h/day), and housework (1.55 h/day). Men spent more time on work, and less on care, childcare, errands, and housework than women. They also reported higher affective wellbeing, and domain-specific life satisfaction but not general life satisfaction. Other gender differences included a higher proportion of full-time work status, and higher education among males.

**Table 1:** Descriptive statistics of sociodemographic data, time use, and wellbeing variables: total, and by gender

		Total				Female				Male				Difference test
		*M* / *n*	*SD* / %	Min	Max	*M* / *n*	*SD* / %	Min	Max	*M* / *n*	*SD* / %	Min	Max	(by gender)
Age (years)		46.87	17.61	18	100	47.12	17.39	18	100	46.60	17.84	18	100	**
Household income (€)		3120.12	2136.49	100	60,000	3102.43	2048.58	150	60,000	3139.20	2227.35	100	60,000	
Number of children in household		0.85	1.25	0	11	0.86	1.24	0	10	0.83	1.26	0	11	**
Gender (1 = female)		15,648	51.90											
Region (1 = East)		6122	20.30			3226	20.62			2896	19.97			
Education	Low	19,068	63.24			10,151	64.87			8917	61.48			
	Medium	3547	11.76			1843	11.78			1704	11.75			
	High	7537	25.00			3654	23.35			3883	26.77			***
Employment	None	14,021	46.50			7829	50.03			6192	42.69			
	Part-time	4897	16.24			3979	25.43			918	6.33			***
	Full-time	11,234	37.26			3840	24.54			7394.00	50.98			***
Marital status (1 = married)		16,586	55.01			8391	53.62			8195	56.50			***
Self-rated Health		3.50	1.01	1	5	3.42	1.00	1	5	3.59	1.01	1	5	***
Life Satisfaction General		7.40	1.75	0	10	7.42	1.76	0	10	7.39	1.74	0	10	†
	Health	6.85	2.29	0	10	6.72	2.30	0	10	7.00	2.27	0	10	***
	Sleep	6.70	2.27	0	10	6.48	2.33	0	10	6.93	2.17	0	10	***
	Free time	7.06	2.16	0	10	6.99	2.22	0	10	7.15	2.09	0	10	***
	Family	7.97	1.87	0	10	7.94	1.88	0	10	8.01	1.86	0	10	**
Affective wellbeing		14.69	2.73	4	20	14.26	2.82	4	20	15.15	2.55	4	20	***
Work		4.60	4.40	0	21	3.91	4.03	0	21	5.34	4.65	0	17	***
Errands		1.09	0.97	0	16	1.28	1.08	0	16	0.89	0.78	0	16	***
Housework		1.55	1.25	0	23	2.06	1.34	0	23	0.95	0.80	0	10	***
Child Care		1.60	3.41	0	24	2.30	4.27	0	24	0.85	1.84	0	24	***
Care		0.18	1.06	0	24	0.23	1.22	0	24	0.12	0.85	0	24	*
Education		0.63	1.87	0	20	0.57	1.79	0	20	0.69	1.95	0	20	
Repairs & garden		0.60	0.89	0	12	0.47	0.77	0	12	0.74	0.98	0	10	
Physical activity		0.64	0.77	0	12	0.60	0.73	0	10	0.68	0.82	0	12	
Leisure		1.77	1.84	0	16	1.72	1.76	0	15	1.83	1.92	0	16	
*N / n*		30,152				15,648				14,504				

*Note.* Test significance: ***p ≤ .001, **p ≤ .01, *p ≤ .05, †*p ≤* .10. *Care*: care and support of persons in need of care; *Leisure*: hobbies and other leisure-time activities

### Latent profiles of daily time-use

**Fig. 1:**
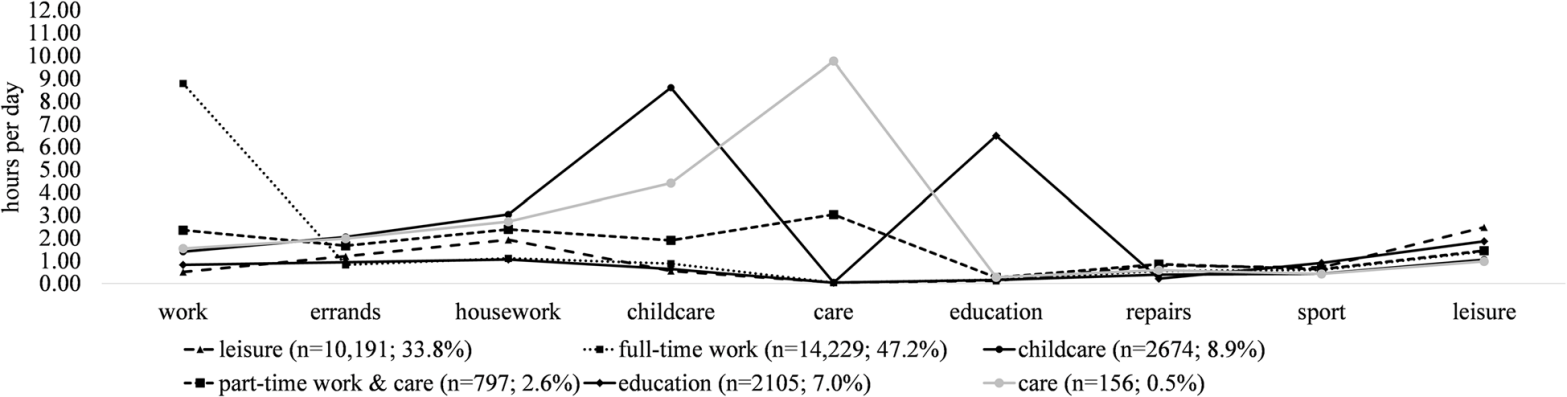
Estimated means and proportions of six latent profiles of daily time use (*n* = 30,152)

Model fit criteria for models with up to six latent profiles are printed in Table 2. Apart from entropy, which was high for all models, the criteria pointed to a solution with six latent profiles as the best fitting model with sufficient theoretical differentiation (see Fig. 1). Although the sixth latent profile was rather small (0.5% of the sample), it seemed to represent persons with intensive home care duties and fewer work hours per day. This profile might reflect informal caregivers, who report worse mental health than non-carers and thus are of relevant target groups for prevention [24, 25]. Therefore, we chose the model with six latent profiles for further analyses. Descriptive statistics of time-use variables for each profile are also printed in a supplementary table (see Additional file 1). Sensitivity analyses (see supplementary tables and figures (Additional file 4)) supported this decision, as a very similar six-profile structure was identified as the best fitting model in persons between 18 an 65 years of age, albeit with slight changes in latent class probabilities. In the older group (66+ years), a four-profile model was preferred, with one very large “leisure” profile (about 93% of the sample) that was very similar to the first profile (named “leisure”) in the total sample. The remaining three profiles resembled the profiles work, education, and care.

**Table 2:** Model fit criteria for latent profile analysis of time use during a typical workday (*n* = 30,152)

	2 profiles	3 profiles	4 profiles	5 profiles	6 profiles
Free parameters	28	38	38	58	68
BLRT	32,017.91***	18,376.54***	29,850.65***	20,304.16***	19,455.63***
AIC	905,412.92	872,886.92	843,056.27	822,772.11	**803,336.47**
SSABIC	905,556.73	873,082.09	843,302.80	823,069.99	**803,685.72**
Entropy	**1.00**	**1.00**	0.97	0.96	0.97
ALCP	1.00	1.00	0.99	1.00	0.97
	0,998	1.00	0.98	0.95	0.98
		1.00	0.98	0.98	0.95
			0.99	0.97	0.99
				1.00	1.00
					1.00

*Note*. *BLRT* Bootstrapped likelihood ratio test, *AIC* Akaike Information Criterion, *SSABIC* Sample-size-adjusted Bayes Information Criterion, *ALCP* Average latent class probabilities; ***p < .001; fit criteria indicating the best model are printed in bold

The first profile “leisure” (*n* = 10,191; 33.8%) was characterized by the highest average leisure time (approximately 2.5 h). The second profile “full-time work” (*n* = 14,229; 47.2%) had an average work investment of about 9 h per day, and less than 1.5 h for all other activities. The third profile “childcare” (*n* = 2674; 8.9%) had a similarly high investment in childcare, but also 2–3 h per day for errands and housework. The fourth profile “part-time work & care” (*n* = 797; 2.6%) had the second highest amount of work hours per day, with an equally high amount of housework and slightly more care duties. The fifth profile “education” (*n* = 2105; 7.0%) dedicated about 6.5 h to educational purposes each day. The sixth profile “care” (*n* = 156; 0.5%) was defined by a high investment in care (about 10 h per day), followed by childcare (about 4.4 h) and housework (about 2.7 h). Across profiles, the average daily time investment for repairs (between 0.21 and 0.85 h per day) and sport/physical activity (between 0.42 and 0.90 h per day) was low.

Descriptive analyses of latent profiles (Table 3) showed that all latent profiles differed in sociodemographic data, self-rated health, life satisfaction, and affective wellbeing. For instance, participants with the profile “education” were significantly younger than all others, while participants in the “childcare” profile lived with an average of 2.42 children in their household. The profile “care” had the lowest scores on almost all indicators of life satisfaction, and affective wellbeing, and profile membership was significantly higher among women and persons with lower education. The profile “leisure” seemed to encompass many persons that were retired or not working, as indicated by its high average age, low household income, and poor education.

**Table 3:** Sociodemographic data, life satisfaction, and affective wellbeing across six latent profiles of daily time use (*n* = 30,152)

		Leisure		Full-time work		Childcare		Part-time work & care		Education		Care	
		*M / n*	*SD / %*	*M / n*	*SD / %*	*M / n*	*SD / %*	*M / n*	*SD / %*	*M / n*	*SD / %*	*M / n*	*SD / %*
Age (years)		58.64	18.75	43.24	12.60	36.41	8.53	52.03^a^	16.13	25.54	9.33	50.38^a^	17.45
Household income (€)		2491.82	1779.29	3711.54	2216.39	2768.42^a^	2013.76	2722.50^a^	1590.04	2780.31^a^	2441.84	2864.89^a^	1535.95
Number of children in household		0.48	1.09	0.78	1.07	2.42	1.35	1.04	1.49	0.88	1.28	1.62	1.85
Gender	(1 = female)	5365	52.64	6330^a^	44.49	2335	87.32	539^b^	67.63	967^a^	45.94	112^b^	71.79
Region	(1 = East)	2215^a^	21.73	2980^a^	20.94	437^b^	16.34	175^a^	21.96	285	13.54	30^a, b^	19.23
Education	Low	7221	70.86	7771	54.61	1667	62.34	555	69.64	1772	84.18	121	77.56
	Medium	831^a^	8.15	2207	15.51	285^b^	10.66	86^b^	10.79	123^c^	5.84	15^a, b, c^	9.62
	High	2139^a^	20.99	4251	29.88	570^a^	21.32	156^a, b^	19.57	401^b^	19.05	20	12.82
Employment	None	9184^a^	90.12	248	1.74	2027^b^	75.80	538	67.50	1900^a^	90.26	124^b^	79.49
	Part-time	883^a^	8.66	3142^b^	22.08	568^b^	21.24	136	17.06	151^c^	7.17	17^a, c^	10.90
	Full-time	124^a^	1.22	10,839	76.18	79^b^	2.95	123	15.43	54^a, b^	2.57	15	9.62
Marital status	(1 = married)	5953^a^	58.41	7870	55.31	1919^b^	71.77	528^c^	66.25	210	9.98	106^a, b, c^	67.95
Self-rated Health		3.17^a^	1.07	3.63^b^	0.89	3.68^b^	0.99	3.28^c^	1.04	4.05	0.94	3.15^a, c^	0.98
Life Satisfaction	General	6.21^a^	2.51	7.10^b^	1.99	7.23^b^	2.31	6.42^a^	2.49	8.01^b^	2.01	6.24^a^	2.40
	Health	6.49^a, b^	2.42	6.84^b^	2.12	6.37^b^	2.31	6.33^a, c^	2.40	7.37	2.07	5.46^b, c^	2.47
	Sleep	7.68^a^	2.04	6.80	2.06	6.30^b^	2.35	6.24^a, b^	2.45	7.28	2.08	5.31^a^	2.61
	Free time	7.95	1.97	7.93	1.81	8.27^a^	1.79	7.69^a^	2.06	8.06	1.81	7.62	1.94
	Family	7.15^a^	1.98	7.56^a^	1.53	7.56	1.81	7.15^b^	1.85	7.53^c^	1.70	6.94^a, b, c^	1.86
Affective wellbeing		14.52	2.87	14.91^a^	2.58	14.28	2.80	13.41^b^	2.95	14.90^a^	2.65	13.41^b^	3.04
*N*		10,191		14,229		2674		797		2105		156	

*Note*. ^*a-c*^ Means in a row without a common superscript letter differ (*p* < 0.05), as analysed by one-way ANOVA with Tukey HSD post-hoc test

### Multinomial logistic regression models

MLRs of time use largely confirmed the differences between profiles regarding sociodemographic variables, revealed several associations of psychological wellbeing and time use profiles, and pointed to additional effects of gender, when using full-time work as a reference group (see Table 4 and supplementary tables (Additional files 2 and 3)). In addition to sociodemographic associations, self-rated health was higher in the part-time work & care profile and the education profile than in the full-time work profile. General life satisfaction was lower in the leisure profile, as was health-related life satisfaction in the childcare profile. In contrast, sleep-related life satisfaction was higher in the leisure profile, and satisfaction with free time was significantly higher in the part-time work & care profile, while at the same time, satisfaction with family life was lower.

**Table 4:** Multinomial logistic regression of latent profiles with full-time work as a reference group (*n* = 30,152)

Class comparison:	Leisure			Childcare			Part-time work & care			Education			Care		
	OR	LB	HB	OR	LB	HB	OR	LB	HB	OR	LB	HB	OR	LB	HB
Age (years)	**1.05**	**[1.04,**	**1.06]**	**0.97**	**[0.96,**	**0.97]**	**1.05**	**[1.04,**	**1.05]**	**0.90**	**[0.90,**	**0.91]**	**1.05**	**[1.04,**	**1.07]**
Household income (€)	**0.87**	**[0.83,**	**0.91]**	**0.82**	**[0.78,**	**0.87]**	**0.87**	**[0.80,**	**0.94]**	**0.96**	**[0.93,**	**0.99]**	0.97	[0.90,	1.04]
Number of children in household	0.97	[0.92,	1.03]	**2.10**	**[1.99,**	**2.22]**	**1.33**	**[1.23,**	**1.44]**	1.04	[0.97,	1.12]	**1.71**	**[1.50,**	**1.95]**
Gender (1 = female)	1.19	[0.59,	2.40]	**3.74**	**[1.11,**	**12.59**	**3.50**	**[1.25,**	**9.79]**	2.13	[0.89,	5.08]	4.33	[0.65,	28.71
Region (1 = East)	**0.64**	**[0.56,**	**0.73]**	**0.65**	**[0.55,**	**0.76]**	**0.79**	**[0.65,**	**0.97]**	**0.57**	**[0.47,**	**0.68]**	0.75	[0.49,	1.14]
Education status Low															
Medium	0.85	[0.72,	1.00]	**1.30**	**[1.08,**	**1.57]**	0.98	[0.76,	1.27]	1.01	[0.81,	1.27]	0.77	[0.43,	1.37]
High	**0.68**	**[0.59,**	**0.77]**	**1.22**	**[1.04,**	**1.42]**	**0.71**	**[0.57,**	**0.88]**	**1.37**	**[1.16,**	**1.62]**	**0.44**	**[0.26,**	**0.74]**
Employment status Not															
Part-time	**0.02**	**[0.02,**	**0.02]**	**0.04**	**[0.03,**	**0.04]**	**0.03**	**[0.03,**	**0.04]**	**0.03**	**[0.02,**	**0.04]**	**0.02**	**[0.01,**	**0.04]**
Full-time	**0.00**	**[0.00,**	**0.00]**	**0.01**	**[0.01,**	**0.01]**	**0.01**	**[0.01,**	**0.02]**	**0.01**	**[0.01,**	**0.01]**	**0.01**	**[0.00,**	**0.02]**
Marital status (1 = married)	**1.36**	**[1.20,**	**1.55]**	**2.38**	**[2.02,**	**2.80]**	**1.68**	**[1.37,**	**2.07]**	0.84	[0.71,	1.00]	**1.61**	**[1.10,**	**2.34]**
Self-rated health	1.29	[0.94,	1.78]	1.27	[0.76,	2.10]	**1.65**	**[1.00,**	**2.73]**	**1.51**	**[1.05,**	**2.18]**	1.11	[0.47,	2.61]
Life satisfaction General	**0.82**	**[0.71,**	**0.94]**	0.93	[0.74,	1.18]	0.90	[0.74,	1.10]	1.13	[0.97,	1.32]	0.86	[0.61,	1.22]
Health	1.01	[0.88,	1.17]	**0.75**	**[0.59,**	**0.95]**	0.87	[0.70,	1.10]	0.97	[0.82,	1.15]	0.85	[0.52,	1.38]
Sleep	**1.16**	**[1.05,**	**1.29]**	1.07	[0.89,	1.29]	1.02	[0.86,	1.21]	0.93	[0.83,	1.05]	1.11	[0.74,	1.65]
Free time	1.00	[0.90,	1.11]	1.16	[0.99,	1.35]	**1.19**	**[1.02,**	**1.38]**	1.01	[0.91,	1.13]	1.19	[0.86,	1.64]
Family	0.95	[0.84,	1.07]	0.91	[0.73,	1.12]	**0.76**	**[0.64,**	**0.90]**	1.02	[0.90,	1.16]	0.78	[0.57,	1.06]
Affective wellbeing	1.04	[0.95,	1.14]	0.94	[0.80,	1.09]	1.06	[0.93,	1.20]	1.01	[0.91,	1.11]	1.08	[0.83,	1.41]
Interactions: Gender X															
Self-rated health	0.89	[0.74,	1.07]	0.95	[0.73,	1.25]	0.77	[0.58,	1.02]	0.86	[0.69,	1.07]	0.92	[0.56,	1.50]
Life satisfaction General	**1.09**	**[0.99,**	**1.19]**	1.08	[0.95,	1.23]	1.06	[0.94,	1.19]	0.96	[0.87,	1.05]	1.08	[0.88,	1.32]
Health	0.96	[0.88,	1.04]	**1.14**	**[1.00,**	**1.29]**	1.07	[0.91,	1.22]	1.01	[0.91,	1.11]	1.15	[0.88,	1.49]
Sleep	1.01	[0.95,	1.07]	0.94	[0.86,	1.04]	1.00	[0.91,	1.10]	1.06	[0.99,	1.14]	0.89	[0.72,	1.11]
Free time	0.98	[0.93,	1.04]	**0.90**	**[0.83,**	**0.98]**	**0.85**	**[0.78,**	**0.93]**	1.00	[0.93,	1.07]	**0.82**	**[0.69,**	**0.97]**
Family	1.01	[0.94,	1.09]	1.06	[0.95,	1.18]	**1.17**	**[1.06,**	**1.29]**	0.97	[0.90,	1.05]	1.18	[0.99,	1.41]
Affective wellbeing	0.97	[0.92,	1.02]	1.02	[0.94,	1.10]	0.95	[0.88,	1.03]	0.99	[0.93,	1.05]	0.93	[0.80,	1.08]

*Note*. *OR* Odds ratio, *LB* Confidence interval lower bound, *HB* Confidence interval higher bound. Significant effects are bolded

Regarding the interactions between gender and psychological wellbeing, findings were mixed with few interactions reaching statistical significance. In most profiles compared to full-time work, women reported lower self-rated health but higher general, health-related and family-related life satisfaction. For these outcomes, three effects were significant, namely higher general life satisfaction in the leisure profile (OR = 1.09 [1.00; 1.19]), higher health-related life satisfaction in the childcare profile (OR = 1.14 [1.00; 1.29]), and higher family-related life satisfaction in the part-time work & care profile (OR = 1.17 [1.06; 1.29]). However, satisfaction with free time was significantly lower for women than men in three profiles (childcare, part-time work & care, and care) when compared to the full-time work profile.

## Discussion

This study investigated latent profiles of daily time use in the general population, and the analysis determined six distinct profiles that were strongly connected to sociodemographic variables, and psychosocial health, which largely mirror previous findings [26]. For instance, women were more likely than men to belong to the profiles named care, childcare, and part-time work & care, whereas persons in the full-time work profile reported higher levels of education. These corroborates previous research on time use in relation to life events (e.g., having children and providing childcare) [24, 37]. In general, persons with a high investment in care were more likely to identify as female, have poorer education, low life satisfaction, and affective wellbeing. Prior research on female caregivers also elucidates childcare and spousal care as particularly detrimental to mental health [38–40]. They are a particularly important group for selective prevention, because they show symptoms of and combine several risk factors for many psychiatric disorders. Future studies could examine cut-offs of daily time spent on care as predictors of worsened mental health and identify thresholds for adaptive preventive interventions. In the current study, participants in the care profile reported an average of 9.76 h of care activities on a typical day. However, the wording of the time use items did not discern time spent on voluntary home care and professional care, therefore responses of “care time” might be inflated by professional working hour estimates. Nevertheless, more attention needs to be put on caregivers as well as care-giving professionals (e.g., nurses) to monitor their levels of activity, and associated mental stress, and wellbeing during the day.

Persons in the leisure profile also had rather low scores in life satisfaction, and self-rated health, but not in respect to domain-specific life satisfaction. The average age of 58.64 years suggests that this profile comprises the elderly for whom declining physical and mental health is expected [41], therefore health-related assessments are less positive. However, we also observed interactions with gender, in that women with higher scores in life satisfaction were more likely to belong to this group. This could indicate that older women have more positive assessments than men, which might be due to different ways of coping (e.g., emotional coping, social support seeking) as suggested by prior research [42].

The profile labelled part-time work and care is particularly noteworthy, because it seems to unite work-related and family-related interests, with higher age, and a female-male ratio of about 2:1. In this profile, wellbeing and health indicators were rather low, despite an overall higher satisfaction with free time compared to the full-time work profile. Yet, differential associations for psychological wellbeing emerged, with women reporting higher satisfaction with family but lower satisfaction with free time than men compared to a full-time working group. This indicates a role conflict, as women may perceive themselves as more family-oriented than men, willing to invest more time into caregiving, but at the same time being challenged to be successful at their (part-time) work, which in turn limits one’s time for leisure activities [21, 24]. While this conclusion is merely hypothetical, and current research points to gender similarities rather than differences concerning the work-family conflict [22], the further distinction of personal free time and family time seems a promising area for future research, as our analyses showed opposing trends for domain-specific life satisfaction. In sum, our findings underscore the potential of time-use surveys to identify personal priorities and add another layer to gender-based comparisons in this area. Future studies could thus provide more inquiry into the life-domain balance of work and family, with regards to role perceptions, by including qualitative and quantitative assessments of domains, time use, roles, and psychosocial wellbeing.

### Strengths, limitations, and future directions

Our study took a holistic approach to adult time-use patterns in comparison to previous studies that only examined specific domains (e.g., physical activity, sedentary behaviours) [5, 10, 11]. We identified distinct profiles of adult time use and described their associations with sociodemographic data, self-rated health, and wellbeing that have implications for future epidemiological research as well as preventive and clinical practice.

However, the study has its limitations. We used a representative data set based on self-reports to capture the subjective experience of time, and personal attitudes. Thus, our results might be affected by recall bias regarding time use measures, and method bias due to a single source. The items mirror previous time use surveys, but they do not clearly discern the context of the activity (e.g., repairs or care duties as part of professional or voluntary work). To combat these limitations, we suggest future studies combine subjective and objective measures, for instance, via digital technologies or ambulatory assessments that allow for an in situ assessment, and a validation of traditional time-use measures. The analysis relied on continuous data, which was limited to 12h hours, but previous studies used more fine-grained measures (e.g., minutes per day) [29, 30], which would allow for a more accurate analysis. As seen in our data, activities like sports or repairs had a low range, and the observed differences might be more meaningful if analysed on a smaller scale.

We interpreted gender differences by presuming that traditional gender roles are still prescribed to [22]. To test this assumption, attitudes towards gender roles should be measured directly and examined in relation to time-use profiles. The current study was cross-sectional and limited to the German population. Latent transition analysis may be used to examine change or stability in time-use patterns and further time-use patterns should be examined across different countries.

## Conclusions

This study identified six latent profiles of daily time use in the general population. These profiles of daily time use closely mirrored sociodemographic aspects, for instance, more time spent on education in younger persons, and more time spent on childcare in middle-aged women with children, which underlines their validity. While most of the sample reported high life satisfaction and wellbeing, a subsample devoted to care had lower scores, indicating a high burden. Moreover, interactions between psychosocial health and gender point to complex domain-specific associations with time use for men and women that warrant further research and point to the importance of actual time use in examining quality of life and psychological wellbeing in epidemiological, population-based inquiry.

## Supplementary Information

**Additional file 1.**

**Additional file 2.**

**Additional file 3.**

**Additional file 4.**

## Data Availability

Codes for the statistical analysis are available upon reasonable request. The datasets generated and/or analysed in the current study are available in the repository of the German Institute for Economic Research, where they will be provided upon request: https://www.diw.de/en/diw_02.c.222843.en/forms.html

## Abbreviations

AIC: Akaike Information Criterion
ALCP: Average latent class probabilities
ANOVA: Analysis of variance
BLRT: Bootstrapped likelihood ratio test
GSOEP: German Socio-Economic Panel
MLRs: Multinomial logistic regression models
OR: Odds Ratio
SSABIC: Sample-size adjusted Bayes Information Criterion

## Footnotes

1 The category “no employment” does not only include persons that are unemployed (i.e. that lost their job and are seeking employment) but also persons who are currently not employed due to other circumstances (e.g., illness or full-time childcare).

## References

[1] Bauman A, Bittman M, Gershuny J (2019). A short history of time use research; implications for public health. BMC Public Health.

[2] Hamermesh DS, Frazis H, Stewart J (2005). Data watch: the American time use survey. J Econ Perspect.

[3] Smith DM. Time use and well-being, and large survey studies. Forum Health Econ Policy. 2011;14(2). 10.2202/1558-9544.1266.

[4] Glorieux I, Laurijssen I, Minnen J, van Tienoven TP (2010). In search of the harried leisure class in contemporary society: time-use surveys and patterns of leisure time consumption. J Consum Policy.

[5] Molina-García J, Castillo I, Queralt A (2011). Leisure-time physical activity and psychological well-being in university students. Psychol Rep.

[6] Smith L, Jacob L, Trott M, Yakkundi A, Butler L, Barnett Y, Armstrong NC, McDermott D, Schuch F, Meyer J, López-Bueno R, Sánchez GFL, Bradley D, Tully MA (2020). The association between screen time and mental health during COVID-19: a cross sectional study. Psychiatry Res.

[7] Virtanen M, Jokela M, Madsen IE, Magnusson Hanson LL, Lallukka T, Nyberg ST (2018). Long working hours and depressive symptoms: systematic review and meta-analysis of published studies and unpublished individual participant data. Scand J Work Environ Health.

[8] Jonsson KR, Oberg G, Samkange-Zeeb F, Adjei NK. Determinants and impact of role-related time use allocation on self-reported health among married men and women: a cross-national comparative study. BMC Public Health. 2020;20(1):1–15. 10.1186/s12889-020-09306-z. 10.1186/s12889-020-09306-z PMC7404928 32758207

[9] Wang S, Mak HW, Fancourt D. Arts, mental distress, mental health functioning & life satisfaction: Fixed-effects analyses of a nationally-representative panel study. BMC Public Health. 2020;20(1):1–9. 10.1186/s12889-019-8109-y. 10.1186/s12889-019-8109-y PMC7014626 32046670

[10] Meyer OL, Castro-Schilo L, Aguilar-Gaxiola S (2014). Determinants of mental health and self-rated health: a model of socioeconomic status, neighborhood safety, and physical activity. Am J Public Health.

[11] Franco OH, Wong YL, Kandala N-B, Ferrie JE, Dorn JM, Kivimäki M, Clarke A, Donahue RP, Manoux AS, Freudenheim JL, Trevisan M, Stranges S (2012). Cross-cultural comparison of correlates of quality of life and health status: the Whitehall II study (UK) and the Western New York health study (US). Eur J Epidemiol.

[12] Gimenez-Nadal JI, Molina JA (2015). Health status and the allocation of time: cross-country evidence from Europe. Econ Model.

[13] Svedberg P, Bardage C, Sandin S, Pedersen NL (2006). A prospective study of health, life-style and psychosocial predictors of self-rated health. Eur J Epidemiol.

[14] Müller G, Tisch A, Wöhrmann AM (2018). The impact of long working hours on the health of German employees. German J Human Res Manage.

[15] Cho S-S, Ju Y-S, Paek D, Kim H, Jung-Choi K (2018). The combined effect of long working hours and low job control on self-rated health: an interaction analysis. J Occup Environ Med.

[16] McKee-Ryan F, Song Z, Wanberg CR, Kinicki AJ (2005). Psychological and physical well-being during unemployment: a meta-analytic study. J Appl Psychol.

[17] Hentschel T, Heilman ME, Peus CV (2019). The multiple dimensions of gender stereotypes: a current look at Men’s and Women’s characterizations of others and themselves. Front Psychol.

[18] van der Meer PH (2014). Gender, unemployment and subjective well-being: why being unemployed is worse for men than for women. Soc Indic Res.

[19] Forret ML, Mainiero LA, Sullivan SE (2010). Gender role differences in reactions to unemployment: exploring psychological mobility and boundaryless careers. J Organiz Behav.

[20] Altweck L, Hahm S, Muehlan H, Gfesser T, Ulke C, Speerforck S, Schomerus G, Beutel ME, Brähler E, Schmidt S (2021). The interplay of gender, social context, and long-term unemployment effects on subjective health trajectories. BMC Public Health.

[21] Sullivan O (2019). Gender inequality in work-family balance. Nat Hum Behav.

[22] Shockley KM, Shen W, DeNunzio MM, Arvan ML, Knudsen EA (2017). Disentangling the relationship between gender and work–family conflict: an integration of theoretical perspectives using meta-analytic methods. J Appl Psychol.

[23] Musick K, Meier A, Flood S (2016). How parents fare. Am Sociol Rev.

[24] Bhan N, Rao N, Raj A (2020). Gender differences in the AssociationsBetween informal caregiving and Wellbeingin low- and middle-income countries. J Women's Health.

[25] Tabler J, Geist C (2021). Do gender differences in housework performance and informal adult caregiving explain the gender gap in depressive symptoms of older adults?. J Women Aging.

[26] Flood SM, Hill R, Genadek KR (2018). Daily temporal pathways: a latent class approach to time diary data. Soc Indic Res.

[27] Liebig S, Goebel J, Schröder C, Grabka M, Richter D, Schupp J (2019). Sozio-oekonomisches Panel (SOEP), Daten der Jahre 1984-2018. SOEP Socio-Economic Panel Study.

[28] Goebel J, Grabka MM, Liebig S, Kroh M, Richter D, Schröder C, Schupp J (2019). The German socio-economic panel (SOEP). Jahrbücher Nationalökonomie Stat.

[29] Schimmack U (2009). Measuring wellbeing in the SOEP. Schmollers Jahr.

[30] Schimmack U, Schupp J, Wagner GG (2008). The influence of environment and personality on the affective and cognitive component of subjective well-being. Soc Indic Res.

[31] R Core Team (2019). R: a language and environment for statistical computing.

[32] van Buuren SG-OK (2011). mice: Multivariate Imputation by Chained Equations in R. J Stat Software.

[33] Muthén LK, Muthén BO (1998). Mplus User’s Guide.

[34] Nylund KL, Asparouhov T, Muthén BO (2007). Deciding on the number of classes in latent class analysis and growth mixture modeling: a Monte Carlo simulation study. Struct Equ Model Multidiscip J.

[35] Tomczyk S, Isensee B, Hanewinkel R (2016). Latent classes of polysubstance use among adolescents—a systematic review. Drug Alcohol Depend.

[36] Asparouhov T, Muthén B (2014). Auxiliary variables in mixture modeling: three-step approaches using M plus. Struct Equ Modeling.

[37] Boll C, Leppin J (2011). Zeitverwendung von Eltern auf Familie und Beruf im internationalen Vergleich.

[38] Penning MJ, Wu Z (2016). Caregiver stress and mental health: impact of caregiving relationship and gender. Gerontologist..

[39] Zick CD, Buder I, Waitzman NJ, Simonsen S, Digre K (2019). The nexus between health and time use among racially and ethnically diverse women. Ethn Health.

[40] Xiong C, Biscardi M, Astell A, Nalder E, Cameron JI, Mihailidis A, Colantonio A (2020). Sex and gender differences in caregiving burden experienced by family caregivers of persons with dementia: a systematic review. PLoS One.

[41] Ryff CD (1991). Possible selves in adulthood and old age: a tale of shifting horizons. Psychol Aging.

[42] Meléndez JC, Mayordomo T, Sancho P, Tomás JM (2012). Coping strategies: gender differences and development throughout life span. Spanish J Psychol.

[43] Infratest Burke Sozialforschung (2018). SOEP-IS – 2018.

