# Supplementary material for: How is the way we spend our time related to psychological wellbeing? A cross-sectional analysis of time-use patterns in the general population and their associations with wellbeing and life satisfaction
Source: BMC Public Health. 2021 Oct 14;21:1858. doi: 10.1186/s12889-021-11712-w (PMC8518234; doi:10.1186/s12889-021-11712-w)
Supplement: Supplementary file 1 — Additional file 1. [file 12889_2021_11712_MOESM1_ESM.docx]

*Supplementary table S1*. Descriptive statistics (means and standard deviations) of daily time use (ranging from 0 to 12+ hours) during a typical workday per latent profile.

|  | Work | Errands | House-work | Child Care | Care | Education | Repairs & garden | Physical activity | Leisure |
| --- | --- | --- | --- | --- | --- | --- | --- | --- | --- |
| Total |  |  |  |  |  |  |  |  |  |
| *M* | 4.58 | 1.09 | 1.55 | 1.48 | 0.17 | 0.62 | 0.60 | 0.64 | 1.77 |
| *SD* | 4.37 | 0.96 | 1.25 | 2.80 | 0.90 | 1.86 | 0.89 | 0.77 | 1.83 |
| Leisure |  |  |  |  |  |  |  |  |  |
| *M* | 0.48 | 1.20 | 1.93 | 0.54 ^a^ | 0.04 ^a^ | 0.12 ^a^ | 0.80 ^a^ | 0.71 | 2.49 |
| *SD* | 1.19 | 0.86 | 1.35 | 1.19 | 0.20 | 0.47 | 1.11 | 0.84 | 2.40 |
| Full-time work |  |  |  |  |  |  |  |  |  |
| *M* | 8.79 | 0.82 | 1.11 ^a^ | 0.87 | 0.04 ^a^ | 0.17 ^b^ | 0.55 ^b^ | 0.59 ^a^ | 1.41 ^a^ |
| *SD* | 1.70 | 0.62 | 0.80 | 1.54 | 0.20 | 0.50 | 0.71 | 0.69 | 1.24 |
| Childcare |  |  |  |  |  |  |  |  |  |
| *M* | 1.38 ^a^ | 2.06 ^a^ | 3.05 | 8.62 | 0.04 ^a^ | 0.16 ^a.b^ | 0.39 ^c^ | 0.42 ^b^ | 1.05 ^b^ |
| *SD* | 2.40 | 1.66 | 1.62 | 2.66 | 0.22 | 0.65 | 0.72 | 0.65 | 1.17 |
| Part-time work & care |  |  |  |  |  |  |  |  |  |
| *M* | 2.37 | 1.67 | 2.37 | 1.89 | 3.03 | 0.26 ^c^ | 0.84 ^a^ | 0.62 ^a^ | 1.44 ^a^ |
| *SD* | 3.48 | 1.40 | 1.38 | 2.76 | 1.26 | 0.88 | 1.18 | 0.99 | 1.52 |
| Education |  |  |  |  |  |  |  |  |  |
| *M* | 0.82 | 0.94 | 1.05 ^a^ | 0.63 ^a^ | 0.03 ^a^ | 6.84 | 0.21 | 0.90 | 1.85 |
| *SD* | 1.98 | 0.89 | 0.84 | 1.61 | 0.21 | 1.91 | 0.50 | 0.87 | 1.63 |
| Care |  |  |  |  |  |  |  |  |  |
| *M* | 1.54 ^a^ | 1.98 ^a^ | 2.71 | 4.40 | 9.76 | 0.28 ^a.c^ | 0.58 ^b.c^ | 0.42 ^b^ | 0.97 ^b^ |
| *SD* | 3.04 | 1.74 | 1.89 | 4.88 | 1.86 | 0.99 | 0.95 | 0.70 | 1.13 |
| *Note*. *^a-c^* Means in a column without a common superscript letter differ (*p* < 0.05), as analysed by one-way ANOVA with Tukey HSD post-hoc test; Care: care and support of persons in need of care; Leisure: hobbies and other leisure-time activities. | | | | | | | | | |
|  |  |  |  |  |  |  |  |  |  |
