# Supplementary material for: How is the way we spend our time related to psychological wellbeing? A cross-sectional analysis of time-use patterns in the general population and their associations with wellbeing and life satisfaction
Source: BMC Public Health. 2021 Oct 14;21:1858. doi: 10.1186/s12889-021-11712-w (PMC8518234; doi:10.1186/s12889-021-11712-w)
Supplement: Supplementary file 2 — Additional file 2. [file 12889_2021_11712_MOESM2_ESM.docx]

#### *Supplementary table S2*. Multinomial logistic regression of latent profiles of daily time use during a typical workday with full-time work as a reference group (*n*=30,152), predicted by sociodemographic data.

| Class comparison: | Leisure | | | | | Childcare | | | | | | | | Part-time work & care | | | | | | | | | | Education | | | | | | | | | | Care | | | | | | | | | |
| --- | --- | --- | --- | --- | --- | --- | --- | --- | --- | --- | --- | --- | --- | --- | --- | --- | --- | --- | --- | --- | --- | --- | --- | --- | --- | --- | --- | --- | --- | --- | --- | --- | --- | --- | --- | --- | --- | --- | --- | --- | --- | --- | --- |
| Full-time work vs. | OR |  | LB | HB |  | OR |  | LB | HB | |  | | OR | |  | | LB | | HB | |  | | OR | |  | | LB | | HB | |  | OR | | |  | LB | | | HB | |  |  |  |
| Age (years) | **1.05** | **[** | **1.05,** | **1.06** | **]** | **0.97** | **[** | **0.96,** | | **0.97** | | **]** | | **1.04** | | **[** | | **1.04,** | | **1.05** | | **]** | | **0.90** | | **[** | | **0.89,** | | **0.91** | | | **]** | **1.05** | | | **[** | **1.04,** | | **1.06** | | | **]** |
| Household income (€) | **0.87** | **[** | **0.83,** | **0.91** | **]** | **0.82** | **[** | **0.78,** | | **0.86** | | **]** | | **0.85** | | **[** | | **0.79,** | | **0.92** | | **]** | | **0.97** | | **[** | | **0.94,** | | **0.99** | | | **]** | 0.93 | | | [ | 0.86, | | 1.01 | | | ] |
| Number of children in household | **0.92** | **[** | **0.87,** | **0.97** | **]** | **2.12** | **[** | **2.01,** | | **2.23** | | **]** | | **1.35** | | **[** | | **1.26,** | | **1.45** | | **]** | | 1.06 | | [ | | 1.00, | | 1.14 | | | ] | **1.77** | | | **[** | **1.57,** | | **1.99** | | | **]** |
| Gender (1=female) | **0.78** | **[** | **0.69,** | **0.89** | **]** | **10.58** | **[** | **8.58,** | | **13.05** | | **]** | | **1.98** | | **[** | | **1.63,** | | **2.40** | | **]** | | 0.91 | | [ | | 0.79, | | 1.05 | | | ] | **2.84** | | | **[** | **1.86,** | | **4.34** | | | **]** |
| Region (1=East) | **0.65** | **[** | **0.57,** | **0.74** | **]** | **0.64** | **[** | **0.54,** | | **0.75** | | **]** | | **0.78** | | **[** | | **0.63,** | | **0.95** | | **]** | | **0.56** | | **[** | | **0.47,** | | **0.67** | | | **]** | 0.70 | | | [ | 0.46, | | 1.07 | | | ] |
| Education status Low |  |  |  |  |  |  |  |  | |  | |  | |  | |  | |  | |  | |  | |  | |  | |  | |  | | |  |  | | |  |  | |  | | |  |
| Medium | **0.85** | **[** | **0.72,** | **0.99** | **]** | **1.33** | **[** | **1.10,** | | **1.59** | | **]** | | 1.00 | | [ | | 0.78, | | 1.29 | | ] | | 1.02 | | [ | | 0.82, | | 1.27 | | | ] | 0.79 | | | [ | 0.44, | | 1.40 | | | ] |
| High | **0.67** | **[** | **0.59,** | **0.77** | **]** | **1.26** | **[** | **1.08,** | | **1.47** | | **]** | | **0.74** | | **[** | | **0.60,** | | **0.91** | | **]** | | **1.42** | | **[** | | **1.20,** | | **1.67** | | | **]** | **0.45** | | | **[** | **0.27,** | | **0.76** | | | **]** |
| Employment status Not |  |  |  |  |  |  |  |  | |  | |  | |  | |  | |  | |  | |  | |  | |  | |  | |  | | |  |  | | |  |  | |  | | |  |
| Full-time | **0.02** | **[** | **0.02,** | **0.02** | **]** | **0.04** | **[** | **0.03,** | | **0.04** | | **]** | | **0.03** | | **[** | | **0.03,** | | **0.04** | | **]** | | **0.03** | | **[** | | **0.03,** | | **0.04** | | | **]** | **0.02** | | | **[** | **0.01,** | | **0.03** | | | **]** |
| Part-time | **0.00** | **[** | **0.00,** | **0.00** | **]** | **0.01** | **[** | **0.01,** | | **0.01** | | **]** | | **0.01** | | **[** | | **0.01,** | | **0.02** | | **]** | | **0.01** | | **[** | | **0.01,** | | **0.01** | | | **]** | **0.01** | | | **[** | **0.01,** | | **0.02** | | | **]** |
| Marital status (1=married) | **1.29** | **[** | **1.14,** | **1.46** | **]** | **2.41** | **[** | **2.05,** | | **2.82** | | **]** | | **1.63** | | **[** | | **1.33,** | | **2.00** | | **]** | | **0.84** | | **[** | | **0.71,** | | **0.99** | | | **]** | **1.56** | | | **[** | **1.07,** | | **2.26** | | | **]** |
| *Note*. OR: odds ratio; LB: confidence interval lower bound; HB: confidence interval higher bound. Significant effects are **bolded**. | | | | | | | | | | | | | | | | | | | | | | | | | | | | | | | | | | | | | | | | | | | |
