# Supplementary material for: How is the way we spend our time related to psychological wellbeing? A cross-sectional analysis of time-use patterns in the general population and their associations with wellbeing and life satisfaction
Source: BMC Public Health. 2021 Oct 14;21:1858. doi: 10.1186/s12889-021-11712-w (PMC8518234; doi:10.1186/s12889-021-11712-w)
Supplement: Supplementary file 3 — Additional file 3. [file 12889_2021_11712_MOESM3_ESM.docx]

#### *Supplementary table S3*. Multinomial logistic regression of latent profiles of daily time use during a typical workday with full-time work as a reference group (*n*=30,152), predicted by sociodemographic data, and psychosocial health variables.

| Class comparison: | Leisure | | | | | Childcare | | | | | | | | Part-time work & care | | | | | | | | | | Education | | | | | | | | | | Care | | | | | | | | | |
| --- | --- | --- | --- | --- | --- | --- | --- | --- | --- | --- | --- | --- | --- | --- | --- | --- | --- | --- | --- | --- | --- | --- | --- | --- | --- | --- | --- | --- | --- | --- | --- | --- | --- | --- | --- | --- | --- | --- | --- | --- | --- | --- | --- |
| Full-time work vs. | OR |  | LB | HB |  | OR |  | LB | HB | |  | | OR | |  | | LB | | HB | |  | | OR | |  | | LB | | HB | |  | OR | | |  | LB | | | HB | |  |  |  |
| Age (years) | **1.05** | **[** | **1.04,** | **1.06** | **]** | **0.97** | **[** | **0.96,** | | **0.97** | | **]** | | **1.04** | | **[** | | **1.04,** | | **1.05** | | **]** | | **0.90** | | **[** | | **0.89,** | | **0.91** | | | **]** | **1.05** | | | **[** | **1.04,** | | **1.07** | | | **]** |
| Household income (€) | **0.86** | **[** | **0.82,** | **0.90** | **]** | **0.83** | **[** | **0.78,** | | **0.87** | | **]** | | **0.87** | | **[** | | **0.80,** | | **0.95** | | **]** | | **0.96** | | **[** | | **0.93,** | | **0.99** | | | **]** | 0.97 | | | [ | 0.91, | | 1.04 | | | ] |
| Number of children in household | 0.97 | [ | 0.92, | 1.02 | ] | **2.09** | **[** | **1.98,** | | **2.21** | | **]** | | **1.30** | | **[** | | **1.21,** | | **1.41** | | **]** | | 1.05 | | [ | | 0.98, | | 1.12 | | | ] | **1.67** | | | **[** | **1.47,** | | **1.91** | | | **]** |
| Gender (1=female) | **0.80** | **[** | **0.70,** | **0.91** | **]** | **10.41** | **[** | **8.43,** | | **12.87** | | **]** | | **1.97** | | **[** | | **1.62,** | | **2.40** | | **]** | | 0.93 | | [ | | 0.80, | | 1.07 | | | ] | **2.71** | | | **[** | **1.78,** | | **4.13** | | | **]** |
| Region (1=East) | **0.64** | **[** | **0.56,** | **0.72** | **]** | **0.64** | **[** | **0.54,** | | **0.76** | | **]** | | **0.79** | | **[** | | **0.64,** | | **0.97** | | **]** | | **0.57** | | **[** | | **0.47,** | | **0.68** | | | **]** | 0.74 | | | [ | 0.49, | | 1.13 | | | ] |
| Education status Low |  |  |  |  |  |  |  |  | |  | |  | |  | |  | |  | |  | |  | |  | |  | |  | |  | | |  |  | | |  |  | |  | | |  |
| Medium | **0.85** | **[** | **0.72,** | **0.99** | **]** | **1.31** | **[** | **1.09,** | | **1.58** | | **]** | | 0.99 | | [ | | 0.76, | | 1.28 | | ] | | 1.01 | | [ | | 0.81, | | 1.26 | | | ] | 0.79 | | | [ | 0.45, | | 1.41 | | | ] |
| High | **0.67** | **[** | **0.59,** | **0.77** | **]** | **1.24** | **[** | **1.06,** | | **1.45** | | **]** | | **0.72** | | **[** | | **0.58,** | | **0.89** | | **]** | | **1.36** | | **[** | | **1.15,** | | **1.61** | | | **]** | **0.45** | | | **[** | **0.27,** | | **0.75** | | | **]** |
| Employment status Not |  |  |  |  |  |  |  |  | |  | |  | |  | |  | |  | |  | |  | |  | |  | |  | |  | | |  |  | | |  |  | |  | | |  |
| Full-time | **0.02** | **[** | **0.02,** | **0.02** | **]** | **0.04** | **[** | **0.03,** | | **0.04** | | **]** | | **0.03** | | **[** | | **0.03,** | | **0.04** | | **]** | | **0.03** | | **[** | | **0.03,** | | **0.04** | | | **]** | **0.02** | | | **[** | **0.01,** | | **0.04** | | | **]** |
| Part-time | **0.00** | **[** | **0.00,** | **0.00** | **]** | **0.01** | **[** | **0.01,** | | **0.01** | | **]** | | **0.01** | | **[** | | **0.01,** | | **0.02** | | **]** | | **0.01** | | **[** | | **0.01,** | | **0.01** | | | **]** | **0.01** | | | **[** | **0.00,** | | **0.02** | | | **]** |
| Marital status (1=married) | **1.36** | **[** | **1.20,** | **1.55** | **]** | **2.38** | **[** | **2.02,** | | **2.79** | | **]** | | **1.68** | | **[** | | **1.36,** | | **2.07** | | **]** | | **0.84** | | **[** | | **0.71,** | | **0.99** | | | **]** | **1.60** | | | **[** | **1.10,** | | **2.34** | | | **]** |
| Self-rated health | 1.08 | [ | 0.99, | 1.18 | ] | **1.18** | **[** | **1.07,** | | **1.31** | | **]** | | 1.07 | | [ | | 0.94, | | 1.23 | | ] | | **1.19** | | **[** | | **1.06,** | | **1.32** | | | **]** | 0.95 | | | [ | 0.75, | | 1.20 | | | ] |
| Life satisfaction General | **0.93** | **[** | **0.90,** | **0.97** | **]** | **1.07** | **[** | **1.02,** | | **1.12** | | **]** | | 0.99 | | [ | | 0.94, | | 1.05 | | ] | | **1.07** | | **[** | | **1.02,** | | **1.12** | | | **]** | 0.97 | | | [ | 0.88, | | 1.07 | | | ] |
| Health | **0.95** | **[** | **0.92,** | **0.99** | **]** | **0.95** | **[** | **0.90,** | | **0.99** | | **]** | | 0.98 | | [ | | 0.93, | | 1.04 | | ] | | 0.97 | | [ | | 0.92, | | 1.02 | | | ] | 1.07 | | | [ | 0.96, | | 1.19 | | | ] |
| Sleep | 1.01 | [ | 0.98, | 1.04 | ] | **0.96** | **[** | **0.92,** | | **0.99** | | **]** | | 1.02 | | [ | | 0.97, | | 1.06 | | ] | | 1.02 | | [ | | 0.98, | | 1.05 | | | ] | **0.91** | | | **[** | **0.83,** | | **0.99** | | | **]** |
| Free time | **1.13** | **[** | **1.10,** | **1.16** | **]** | 0.97 | [ | 0.94, | | 1.00 | | ] | | **1.10** | | **[** | | **1.06,** | | **1.15** | | **]** | | 1.01 | | [ | | 0.97, | | 1.04 | | | ] | **0.85** | | | **[** | **0.78,** | | **0.92** | | | **]** |
| Family | 0.97 | [ | 0.94, | 1.01 | ] | 1.01 | [ | 0.97, | | 1.05 | | ] | | 0.98 | | [ | | 0.93, | | 1.03 | | ] | | 0.98 | | [ | | 0.95, | | 1.02 | | | ] | 1.03 | | | [ | 0.94, | | 1.12 | | | ] |
| Affective wellbeing | 0.99 | [ | 0.97, | 1.02 | ] | **0.97** | **[** | **0.95,** | | **0.99** | | **]** | | 0.97 | | [ | | 0.94, | | 1.01 | | ] | | 0.99 | | [ | | 0.96, | | 1.02 | | | ] | 0.97 | | | [ | 0.90, | | 1.03 | | | ] |
| *Note*. OR: odds ratio; LB: confidence interval lower bound; HB: confidence interval higher bound. Significant effects are **bolded**. | | | | | | | | | | | | | | | | | | | | | | | | | | | | | | | | | | | | | | | | | | | |
