# Supplementary material for: How is the way we spend our time related to psychological wellbeing? A cross-sectional analysis of time-use patterns in the general population and their associations with wellbeing and life satisfaction
Source: BMC Public Health. 2021 Oct 14;21:1858. doi: 10.1186/s12889-021-11712-w (PMC8518234; doi:10.1186/s12889-021-11712-w)
Supplement: Supplementary file 4 — Additional file 4. [file 12889_2021_11712_MOESM4_ESM.docx]

#### **Sensitivity Analysis**

#### This file contains model fit criteria and latent profiles of the sensitivity analysis that differentiated younger (18-65 years) and older (66+ years) participants in our sample. Latent profiles of the younger subsample are quite similar to the original analysis reported in the text (including all participants), while latent profiles of the older subsample differ.

#### *Supplementary table S4.* Model fit criteria for latent profile analysis of time use during a typical workday in a subsample of 18- to 65-year-olds (*n*=25,048).

|  | 2 profiles | 3 profiles | 4 profiles | 5 profiles | 6 profiles |
| --- | --- | --- | --- | --- | --- |
| Free parameters | 28 | 38 | 48 | 58 | 68 |
| BLRT | 32017.91*** | 18376.54*** | 29850.65*** | 20304.16*** | **19455.63***** |
| AIC | 742509.39 | 715383.57 | 690534.23 | 676048.47 | **662677.77** |
| SSABIC | 742648.01 | 715571.69 | 690771.85 | 676335.61 | **663014.41** |
| Entropy | 0.99 | **1.00** | 0.96 | 0.98 | 0.96 |
| ALCP | 1.00 | 1.00 | 0.97 | 0.99 | 0.94 |
|  | 1.00 | 1.00 | 0.98 | 0.95 | 0.94 |
|  |  | 1.00 | 1.00 | 0.99 | 0.99 |
|  |  |  | 0.99 | 1.00 | 0.98 |
|  |  |  |  | 1.00 | 1.00 |
|  |  |  |  |  | 1.00 |
| *Note*. BLRT bootstrapped likelihood ratio test; AIC Akaike Information Criterion; SSABIC sample-size-adjusted Bayes Information Criterion; ALCP average latent class probabilities; ****p*< .001; fit criteria indicating the best model are printed **in bold**. | | | | | |


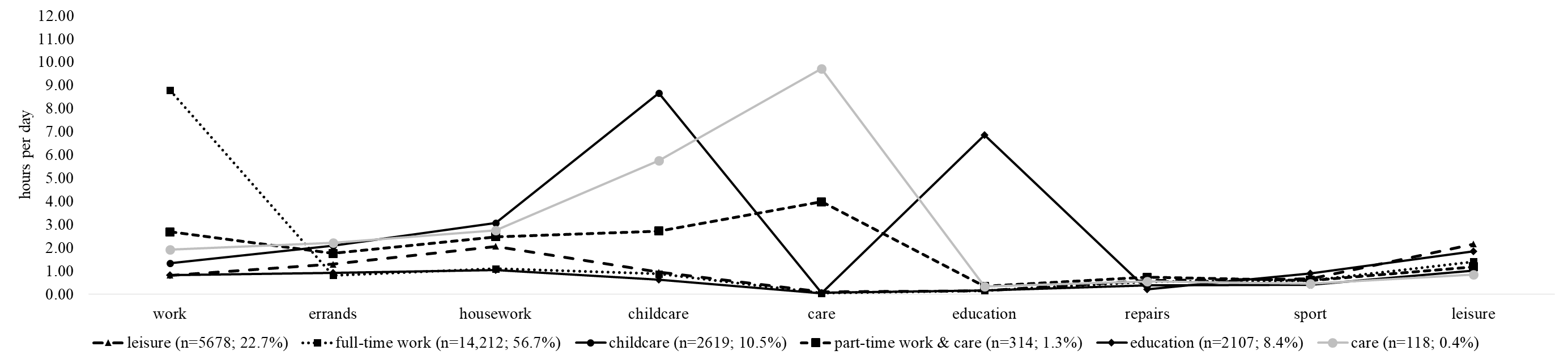


**Supplementary Figure S1**. Estimated means and proportions of six latent profiles of daily time use in a subsample of 18- to 65-year-olds (*n*=25,048)

#### *Supplementary table S5.* Model fit criteria for latent profile analysis of time use during a typical workday in a subsample of 66+-year-olds (*n*=5,104).

|  | 2 profiles | 3 profiles | 4 profiles | 5 profiles | 6 profiles |
| --- | --- | --- | --- | --- | --- |
| Free parameters | 28 | 38 | 48 | Did not converge | Did not converge |
| BLRT | 32017.91*** | 18376.54*** | 29850.65*** |  |  |
| AIC | 122024.59 | 116635.71 | **112513.79** |  |  |
| SSABIC | 122118.67 | 116763.40 | **112675.07** |  |  |
| Entropy | 0.99 | 0.99 | **0.99** |  |  |
| ALCP | 1.00 | 1.00 | 1.00 |  |  |
|  | 1.00 | 1.00 | 1.00 |  |  |
|  |  | 1.00 | 1.00 |  |  |
|  |  |  | 1.00 |  |  |
|  |  |  |  |  |  |
|  |  |  |  |  |  |
| *Note*. BLRT bootstrapped likelihood ratio test; AIC Akaike Information Criterion; SSABIC sample-size-adjusted Bayes Information Criterion; ALCP average latent class probabilities; ****p*< .001; fit criteria indicating the best model are printed **in bold**. | | | | | |


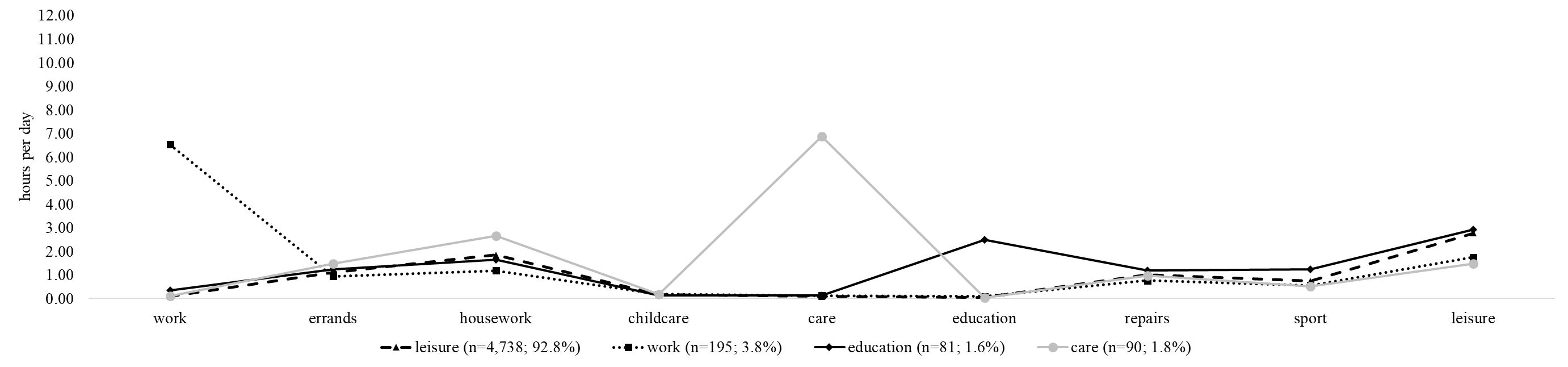


**Supplementary Figure S2**. Estimated means and proportions of six latent profiles of daily time use in a subsample of 66+-year-olds (*n*=5,104)
